# SB-224289 Antagonizes the Antifungal Mechanism of the Marine Depsipeptide Papuamide A

**DOI:** 10.1371/journal.pone.0154932

**Published:** 2016-05-16

**Authors:** Chelsi D. Cassilly, Marcus M. Maddox, Philip T. Cherian, John J. Bowling, Mark T. Hamann, Richard E. Lee, Todd B. Reynolds

**Affiliations:** 1 Department of Microbiology, University of Tennessee, Knoxville, Tennessee, United States of America; 2 Department of Chemical Biology and Therapeutics, St. Jude Children’s Research Hospital, Memphis, Tennessee, 38105, United States of America; 3 Department of Drug Discovery and Biomedical Sciences, College of Pharmacy, Medical University of South Carolina, Medical University of South Carolina, Charleston, South Carolina, United States of America; Louisiana State University, UNITED STATES

## Abstract

In order to expand the repertoire of antifungal compounds a novel, high-throughput phenotypic drug screen targeting fungal phosphatidylserine (PS) synthase (Cho1p) was developed based on antagonism of the toxin papuamide A (Pap-A). Pap-A is a cyclic depsipeptide that binds to PS in the membrane of wild-type *Candida albicans*, and permeabilizes its plasma membrane, ultimately causing cell death. Organisms with a homozygous deletion of the *CHO1* gene (*cho1ΔΔ*) do not produce PS and are able to survive in the presence of Pap-A. Using this phenotype (i.e. resistance to Pap-A) as an indicator of Cho1p inhibition, we screened over 5,600 small molecules for Pap-A resistance and identified SB-224289 as a positive hit. SB-224289, previously reported as a selective human 5-HT_1B_ receptor antagonist, also confers resistance to the similar toxin theopapuamide (TPap-A), but not to other cytotoxic depsipeptides tested. Structurally similar molecules and truncated variants of SB-224289 do not confer resistance to Pap-A, suggesting that the toxin-blocking ability of SB-224289 is very specific. Further biochemical characterization revealed that SB-224289 does not inhibit Cho1p, indicating that Pap-A resistance is conferred by another undetermined mechanism. Although the mode of resistance is unclear, interaction between SB-224289 and Pap-A or TPap-A suggests this screening assay could be adapted for discovering other compounds which could antagonize the effects of other environmentally- or medically-relevant depsipeptide toxins.

## Introduction

Patients with a compromised immune system are prone to develop nosocomial infections which can be fungal, bacterial, parasitic, or viral in nature. *Candida spp*. are pathogenic fungi responsible for the majority of fungal infections arising within hospitals [1, 2]. Of these, the species found most often is *Candida albicans* [3]. This fungus most commonly causes mucosal infections including vaginal infections and oral thrush, but it can also cause life-threatening infections. The most serious of these include fungal endocarditis and systemic bloodstream infections which have mortality rates around or greater than 30% [4, 5].

There are currently three primary classes of antifungals used to treat invasive *Candida* infections. The first line defense includes azoles (e.g. fluconazole) and echinocandins (e.g. caspofungin). However, resistance and tolerance to these drugs can lead to treatment failures [6–9]. The mainstay of second line defense is the polyene amphotericin B, a drug that must be carefully administered due to its nephrotoxic potential [10, 11]. As a result of drug resistance, poor oral availability, and toxic side effects including drug-drug interactions [12], there is a clear need for new antifungal drugs to treat *Candida* infections.

Previously, the fungal phosphatidylserine (PS) synthase (Cho1p) was identified as a promising antifungal drug target for several reasons. First, Cho1p has been demonstrated to be required for *Candida albicans* virulence in a mouse model of systemic infection [13]. Mice infected with a strain of *C*. *albicans* where both alleles of the *CHO1* gene are deleted (*cho1ΔΔ*) were able to survive indefinitely, whereas mice infected with the wild-type strain succumbed to infection within two weeks [13]. Second, the Cho1p enzyme is conserved among many pathogenic fungi [13, 14]. Last, Cho1p is absent within mammals [13–16]. Based on these observations, we hypothesized that high affinity inhibitors of Cho1p should render the organism unable to cause infection within a host, be active against a broad range of fungal pathogens, and be highly selective with minimal side effects in mammals.

Based on this hypothesis, we set out to identify inhibitors of this pathway using a cell based high-throughput screening approach. The assay is based on the action of a PS-dependent toxin, papuamide A (Pap-A), which is a cyclic depsipeptide isolated from marine sponges of the genus *Theonella*. Pap-A kills *C*. *albicans* and other yeasts by binding to PS in the membrane and forming pores that disrupt the integrity of the membrane [17, 18]. We wished to exploit the PS-specific nature of Pap-A toxicity and identify compounds that block PS synthesis or alternatively interfere with PS metabolism, by selecting those small molecules that allow *C*. *albicans* survival in the presence of Pap-A. Thus, the Pap-A killing assay was adapted into a robust 384-well plate screening assay and tested against a reference set of bioactive compounds including many known drugs.

This screen showed a good statistical window and yielded a promising hit. However, we found that Pap-A resistance is not specific enough to conclude that a compound targets Cho1p, as other mechanisms can be responsible for this protection phenotype. In this study we describe the characterization of a positive hit, SB-224289, which in turn showed an interesting and highly specific behavior that blocks Pap-A mediated cellular poisoning.

## Materials and Methods

### Strains used

The SC5314 (wild-type) strain of *C*. *albicans* and mutants used in this study have been previously described [13] and are as follows: *cho1ΔΔ* (YLC337), and *cho1ΔΔ*::*CHO1* (YLC344). The media used to culture strains was YPD (1% Bacto yeast extract, 2% Bacto peptone, and 2% dextrose (Thermo Fisher Scientific, San Jose, CA)) [19].

### Compounds

The 5,760 bioactive compound library is a collated compound set of approved drugs (675 compounds) and biologically active compounds that have been documented to interact with a wide range of targets (approximately 5,095 compounds) including Sigma Aldrich’s Library of Pharmacologically Active Compounds (LOPAC), Prestwick, and Microsource compound libraries. The library compounds were all independently verified for purity and identity by UPLC-MS analysis. Papuamide A was obtained from Flintbox (David Williams and Raymond Andersen, University of British Colombia, Canada). SB-224289 (Cat. # 1221), MG-624 (Cat. # 1356), and valinomycin (VA; Cat. # 3373) were ordered from Tocris Bioscience. GMC 2–29 (Cat. # 1080) and SB-216641 (Cat. # 1085) were ordered from Axon MedChem. Staurosporine (CGP 41251) was ordered from Selleckchem. Kahalalide F (KF) was a kind gift from Dr. Fernando Albericio and Gerardo Acosta at the Institute for Research in Biomedicine, Barcelona, Spain. Theopapuamide (TPap-A) was provided by Dr. Mark Hamann at University of Mississippi, University, Mississippi, USA. Compounds 2945, 2946, 3047, and 3048 were synthesized as described previously [20], and details of the synthesis and compound characterization are provided in the Supporting Information (S1 File).

### Papuamide A resistance assay

Strains were grown overnight in liquid YPD shaking at 30°C to saturation, and cultures were diluted to 2 x 10^4^ cells/ml in YPD. Compounds of interest were diluted to twice the working concentration by serial dilution in a 96 well plate or by preparing separately and adding to the wells directly, in a volume of 37.5 μl of YPD. Then 37.5 μl of cells at 2 x 10^4^ cells/ml in YPD were added. Plates were incubated at 37°C for 6 hours or 3 hours depending on the experiment, and then 75 μl of YPD containing depsipeptide (Pap-A at 8 μg/ml, VA at 6 μg/ml, KF at 30 μg/ml, or TPap-A at 12 μg/ml) were added to each well, diluting those concentrations by half. This addition was followed by a 37°C overnight incubation.

Cell survival was measured the next day by fluorescence intensity or optical density. For fluorescence intensity, Alamar Blue (Invitrogen, Waltham, MA) was added to the wells at a 1:10 dilution. Plates were allowed to incubate again at 37°C for 30 minutes to 2 hours until color change was apparent. Fluorescence was then read at excitation 550 nm and emission 590 nm. For optical density, plates were removed from overnight incubation and absorbance was read in a plate reader at a wavelength of 600 nm. All measurements were performed on a Cytation3 BioTek plate reader using Gen 5 software.

### High throughput screen for Pap-A resistance

The 5,760 compound library was screened in a total of eighteen 384 well plates (Nunc) at a final concentration of 50–75 μM. Approximately 0.155 μl of compound dissolved in DMSO were inoculated into 10 μl of YPD in each well from ~10 mM stock plates using a BioMek robot with pin tools. Wild-type and *cho1ΔΔ* (positive control strain) were grown in liquid YPD in a 30°C shaker overnight and cultures were diluted to 10^4^ cells/ml in YPD.10 μl were added to each well of the 384 well plate containing the test compounds using a Wellmate. Plates were incubated for 6 hours at 37°C, and then 10 μl of YPD containing 12 μg/ml Pap-A was added to give a final concentration of 4 μg/ml in 30 μl of YPD. Plates were then incubated for another 16 hours at 37°C to allow selection for Pap-A resistance. The following day, cell survival was measured by adding 30% Alamar Blue (Invitrogen, Waltham, MA) using a Wellmate. Plates were allowed to incubate again at 37°C for 2–3 hours. Fluorescence was then read at excitation 550 nm and emission 590 nm on an Envision plate reader (Perkin Elmer, Waltham, MA). In each plate, three of the columns of wells were used as controls. One column contained no compounds and no cells and served as background control. The positive control column contained *cho1ΔΔ* plus Pap-A, as it showed resistance. An additional control was wild-type with no drugs and no Pap-A for maximal wild-type growth. The averaged background readings from the wild-type cells plus compounds in the presence of Pap-A served as the negative control in each plate.

### Phosphatidylserine synthase assay

This procedure was done as described in [21, 22] with minor alterations. Cultures were grown overnight and then diluted to approximately 0.1 OD_600_/ml in 1 L YPD, and were shaken at 30°C for 6 to 10 hours. Cells were harvested by centrifugation at 6,000 x g for 20 minutes. Pellets were then transferred to 50 ml conical tubes and washed with water and re-pelleted. Supernatant was removed and the wet weight of the samples was taken. Cell pellets were stored overnight at -80°C. The following day, a cold mixture of 0.1 M Tris-Cl pH 7.5, 5 mM β-mercaptoethanol (BME), 10% glycerol, and protease inhibitors 1.7 μg/ml PMSF, 1 μg/ml leupeptin, and 1 μg/ml pepstatin (RPI, Corp., Mount Prospect, IL, USA) was added to the frozen pellets (1 ml/g [wet weight]) and allowed to thaw on ice. Cells were lysed using a French press (three passes at approximately 13,000 lb/in^2^). The homogenate was centrifuged at 4°C for 5 minutes at 3,000 rpm to clear unbroken cells and heavy material. Supernatant was then spun again at 27,000 x g for 10 minutes at 4°C. For some experiments, the resulting supernatant was then spun at 100,000 x g to collect the lower density membranes. Pellets were resuspended in 500 μl to 1 ml of 0.1 M Tris-Cl pH 7.5, 5 mM BME, 10% glycerol, and protease inhibitors. This mixture was aliquoted into microcentrifuge tubes and homogenized to break apart clumps, keeping on ice as much as possible. Total crude protein concentration was determined using a Bradford Assay. The optimal assay mixture contained 50 mM Tris-HCl pH 7.5, 0.1% Triton X-100, 0.5 mM MnCl_2_, 0.1 mM CDP-DAG (Avanti Polar Lipids, Alabaster, AL) added as a suspension in 1% - 20% Triton X-100, and 0.4–0.5 mg protein in a total volume of 0.1 ml. SB-224289 and MG-624 were added to the reaction mixture at varying concentrations to monitor their ability to inhibit [^3^H]-PS production. The PS synthase assay was performed by monitoring the incorporation of 0.5 mM l-serine spiked with 5% [^3^H]-l-serine (or 0.02 μM) (Cat# ART 0246, ARC, Inc., St. Louis, MO, USA) into the chloroform-soluble product at 37°C for a predetermined amount of time. The reaction was terminated by the addition of 1 ml chloroform: methanol (2:1). Following a low-speed spin, 800–1000 μl of the supernatant was removed to a fresh tube and washed with 200 μl 0.9% NaCl. Following a second low-speed spin, 400–500 μl of the organic phase was removed to a new tube and washed with 500 μl of chloroform: methanol: 0.9% NaCl (3:48:47). Following a third low-speed spin, 200–300 μl was transferred into scintillation vials (Thermo Fisher Scientific, San Jose, CA). Tubes were dried under the chemical hood and 2.5 ml Cytoscint-ES liquid scintillation cocktail (MP Bio, Santa Ana, CA, USA) was added to each tube and counted in a Packard TriCarb 2900TR Liquid Scintillation Analyzer.

### Statistical analysis

Graphs were made using GraphPad Prism version 6.04. Unpaired t-tests were used to determine significance between results. RISE (Robust Investigation of Screening Experiments) software was used to analyze data from the high throughput screen and was used to calculate Z-factors, identify 95^th^ and 99^th^ quantile data, and identify compounds that yielded hits of greater than 90% of positive control (*cho1ΔΔ*).

## Results

### Screen for compounds that confer Pap-A resistance

**Fig 1:**
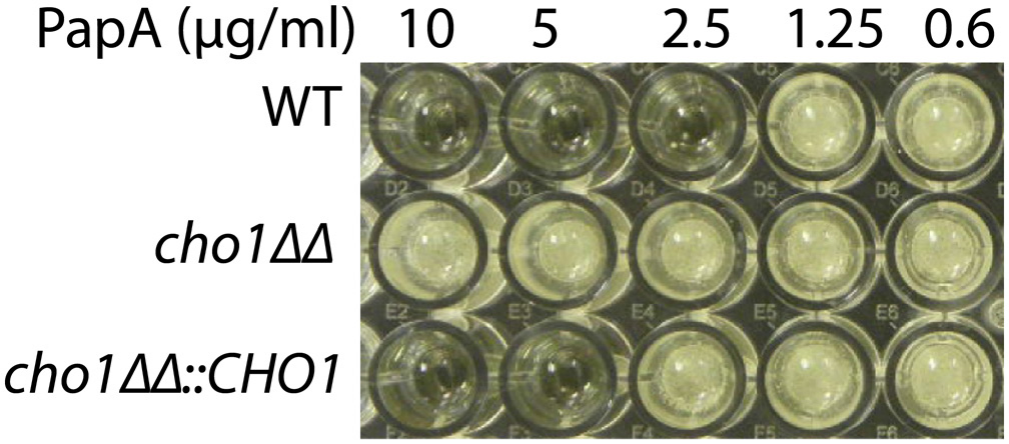
Resistance to Pap-A correlates with decreases in PS. The *cho1ΔΔ* mutant is resistant to all concentrations of papuamide A (Pap-A) indicating a total lack of PS. The *cho1ΔΔ*::*CHO1* reintegrant strain is more resistant to Pap-A than the wild-type (WT), but less resistant than the *cho1ΔΔ* mutant.

A novel screen was developed with an original goal of identifying compounds that inhibit the Cho1p PS synthase enzyme. This screen utilizes the unique activity of Pap-A, a toxin isolated from *Theonella spp*. of sponges [23]. Pap-A has been shown to selectively compromise the integrity of membranes containing PS [17, 18]. As a result, Pap-A is toxic to wild-type *C*. *albicans*. However, the *cho1ΔΔ* mutant, which lacks Cho1p, and thus has no PS in its membranes, is able to survive in the presence of Pap-A (Fig 1) [13]. A reintegrated strain, *cho1ΔΔ*::*CHO1*, which has one allele of *CHO1* and intermediate levels of PS [13], shows intermediate resistance (Fig 1). This feature of Pap-A was employed as a selection tool in our screen to identify those compounds that inhibit Cho1p. We hypothesized that some compounds that allow wild-type growth in the presence of Pap-A would do so by decreasing levels of PS in membranes.

The screen was performed on approximately 5,600 diverse pharmacologically active compounds as a representative set of chemical space. Each plate contained a column for each control: 1) *cho1ΔΔ* + Pap-A as a positive control 2) no cells/no Pap-A as a background control 3) wild-type cells with no drugs or Pap-A for a wild-type growth control. The remainder of the plate was reserved for compound testing by pin transfer, and served as a control for background of wild-type cells killed by Pap-A.

**Fig 2:**
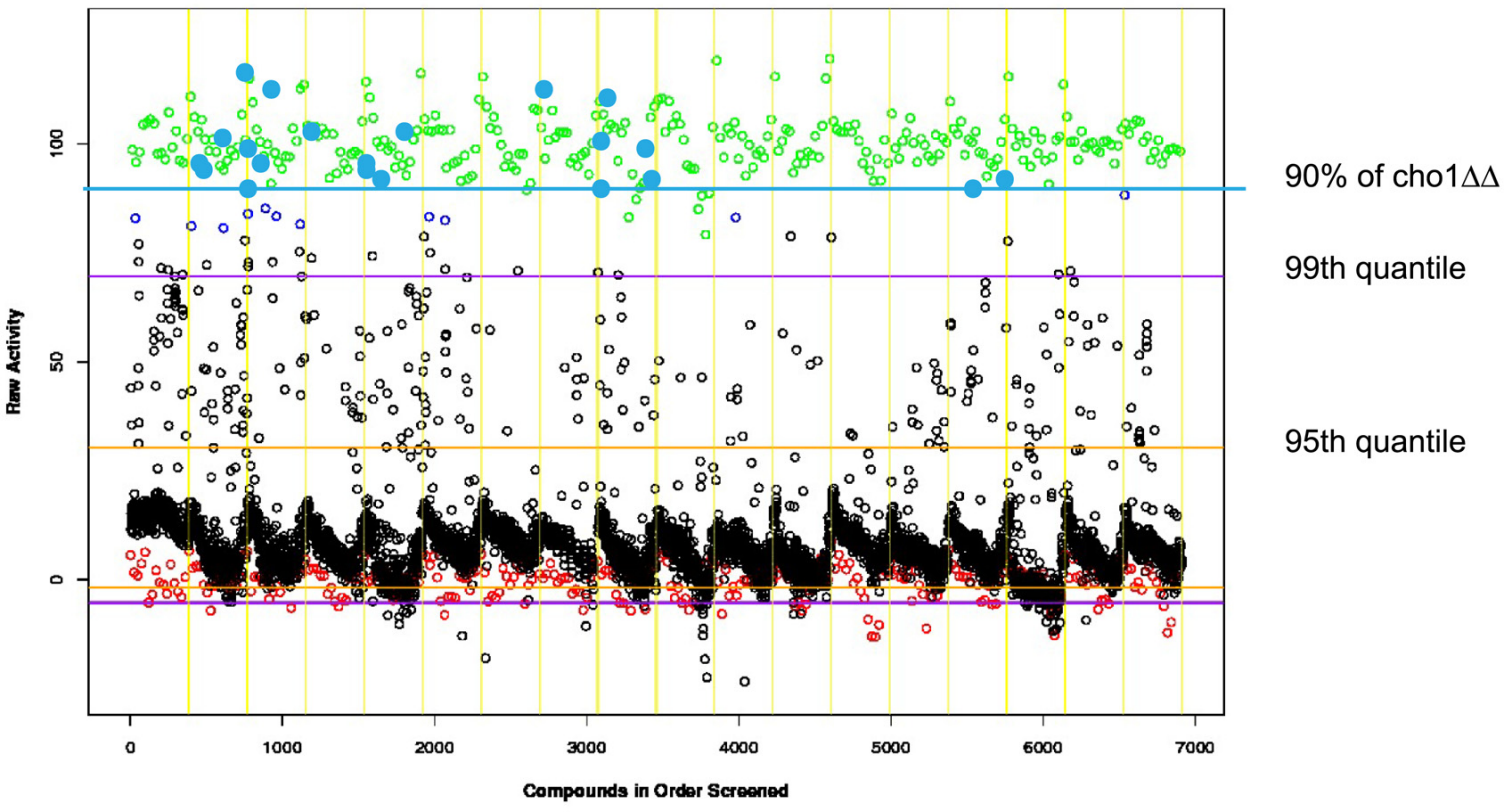
Screen of FDA-approved bioactive compounds for those that confer Pap-A resistance. 5,760 compounds were screened for their effects on the growth of wild-type *C*. *albicans* (open black circles) in the presence of 4 μg/ml Pap-A. Cell growth was measured by transformation of the dye Alamar Blue over approximately 3 hours at 37°C. The *cho1ΔΔ* positive control cells growth in the presence of Pap-A with no compounds from the library are represented by green circles. Compounds that allowed wild-type cells to display >90% (above the blue line) of the growth of *cho1ΔΔ* control were designated with filled-in blue circles. Around 95% of the tested compounds showed growth levels closer to the negative control, wells which contained no cells or drugs (open red circles). The horizontal lines show the 99th quantile (purple) where 99% of the compounds exhibited growth and the 95th quantile (yellow) 95% of the compounds lie. The vertical lines divide the compounds by the 384-well plate in which they were screened which correlate to plate numbers along the bottom. A full description of the screening method is found in Materials and Methods.

**Fig 3:**
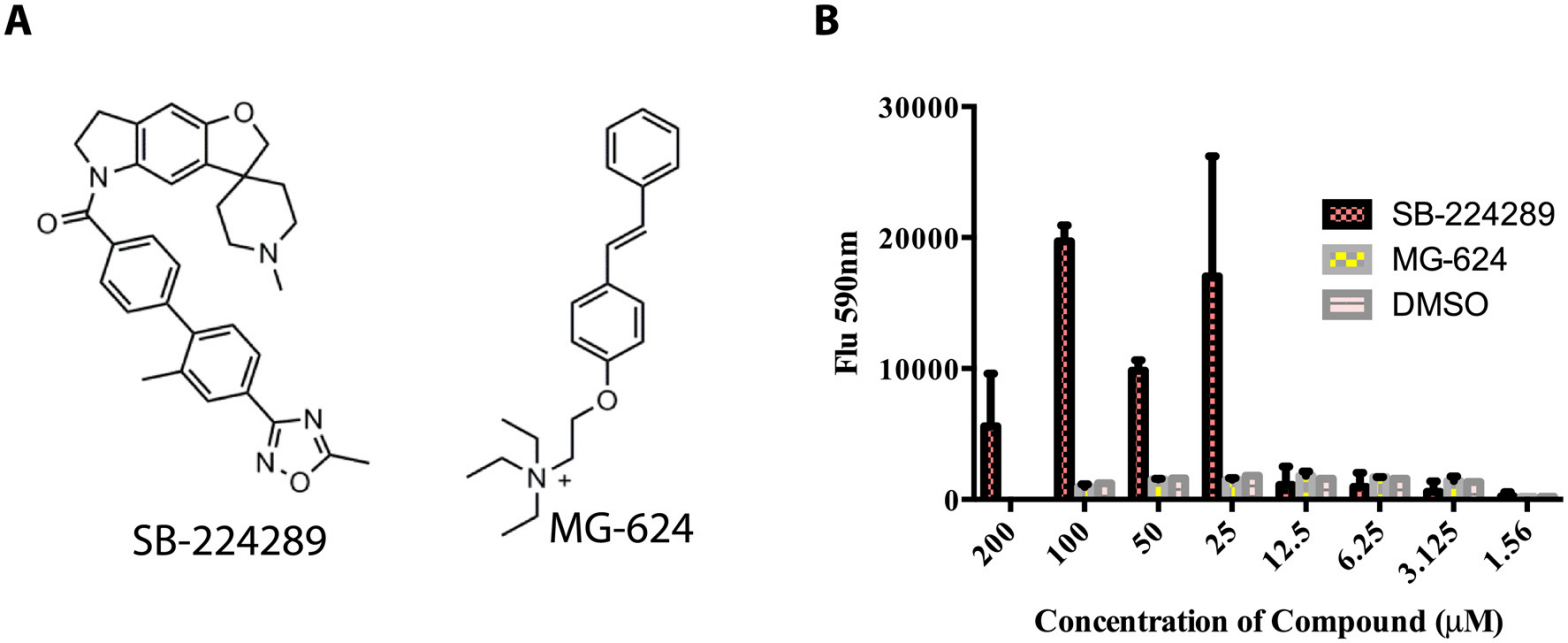
Compounds that conferred Pap-A resistance to *C*. *albicans* wild-type yeast. A) Structures of hits identified from the screen. B) Comparison of the ability of the hits to confer Pap-A resistance to wild type *C*. *albicans;* SB-224289, MG-624, and DMSO as a control.

The majority of test wells (black open circles in Fig 2), which contained cells that were killed by Pap-A, read near the no drug/no cell background control (red circles, Fig 2). Compounds were tested at a final concentration of 50–75 μM for their ability to confer resistance to wild-type *C*. *albicans* against a lethal dose of Pap-A (4 μg/ml). After incubation overnight, the viability indicator Alamar Blue was added and fluorescence was measured at 590 nm as a proxy for survival. Of the tested compounds, 21 (filled in blue circles) showed viability greater than or equal to 90% of the *cho1ΔΔ* positive control (green circles) (Fig 2). These 21 compounds, were considered first round positive hits and were selected and tested for reproducibility in providing Pap-A resistance. Of these 21 first round hits, two compounds reproducibly conferred Pap-A resistance to the wild-type cells: SB-224289, a serotonin receptor antagonist [24], and MG-624, a nicotinic acetylcholine receptor antagonist (Fig 3A).

Dose response curves were performed to determine the lowest concentrations of SB-224289 and MG-624 that could confer Pap-A resistance. Serial dilutions starting at 200 μM revealed that SB-224289 could confer resistance down to 25 μM, while MG-624 was not consistently effective at producing Pap-A resistance. We found that effects of SB-224289 at concentrations above 400 μM could not be determined as this was greater than its solubility limit in the test media. Efforts were focused on SB-224289, which showed consistent efficacy at a concentration range from 100 μM to 25 μM (Fig 3B).

### Identification of structural features that enable SB-224289 to confer Pap-A resistance

**Fig 4:**
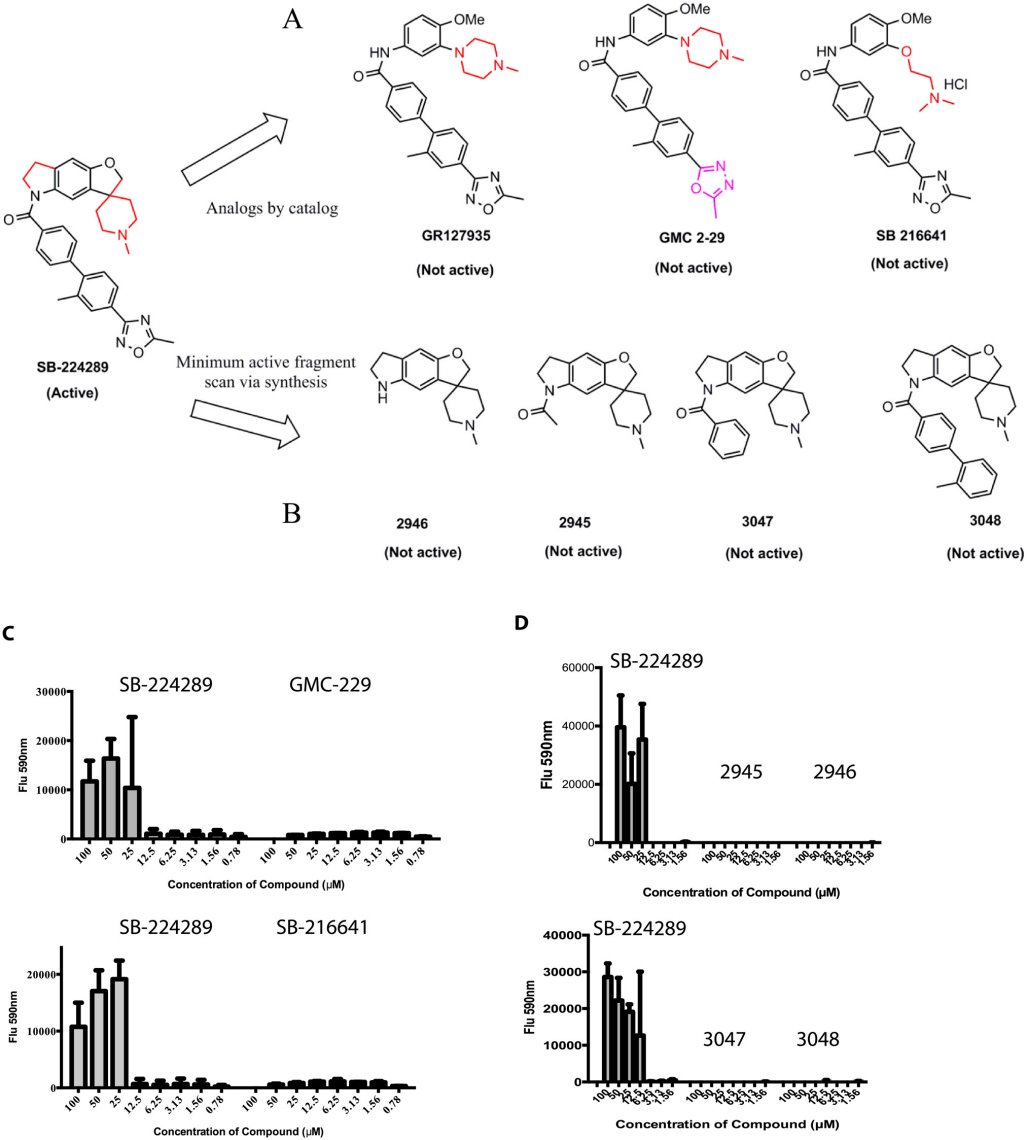
The full structure of SB-224289 is required to provide Pap-A resistance. **(A)** and **(B)** show the structures of purchased or synthesized analogs of SB-224289, respectively. In **(C)** and **(D)** 1x10^4^ cells/ml were treated with a serial dilution of SB-224289, test compounds or DMSO control, and then allowed to incubate for 6 hours at 37°C. Pap-A was added at either 4 μg/ml or 5 μg/ml after 6 hours the plate was incubated at 37°C overnight. Alamar Blue was added and fluorescence signal measured at 590 nm indicating survival of cells.

In order to determine if Pap-A resistance is a property common to compounds with a similar structure to SB-224289, we searched the other compounds screened in our library and found one structural analog, GR-127935, which did not provide Pap-A resistance (Fig 4A). Two other structural analogs of SB-224289, GMC 2–29 and SB-216641, were tested to assess their ability to confer Pap-A resistance to wild-type *C*. *albicans* (Fig 4A and 4C). We found that neither analog rescued cells when compared with the SB-224289 control. This finding prompted us to hypothesize that the areas of structural dissimilarity between SB-224289 and GR-127935, GMC 2–29, and SB-216641—specifically the spirofuro-indole ring structure unique to SB-224289—may be the key to conferring Pap-A resistance. We synthesized four SB-224289 ring structure homologues, 2945, 2946, 3047, and 3048 (Fig 4B and S1 File), and tested their ability to provide Pap-A resistance to wild-type *C*. *albicans*. Although we expected protection against Pap-A at similar or even lower concentrations, none of the synthesized SB-224289 ring structures provided resistance to Pap-A (Fig 4B and 4D). These data indicate that the full molecular structure of SB-224289 is required for producing Pap-A resistance to wild-type *C*. *albicans*.

### Exploration of the Mechanism of Action of SB-224289

**Fig 5:**
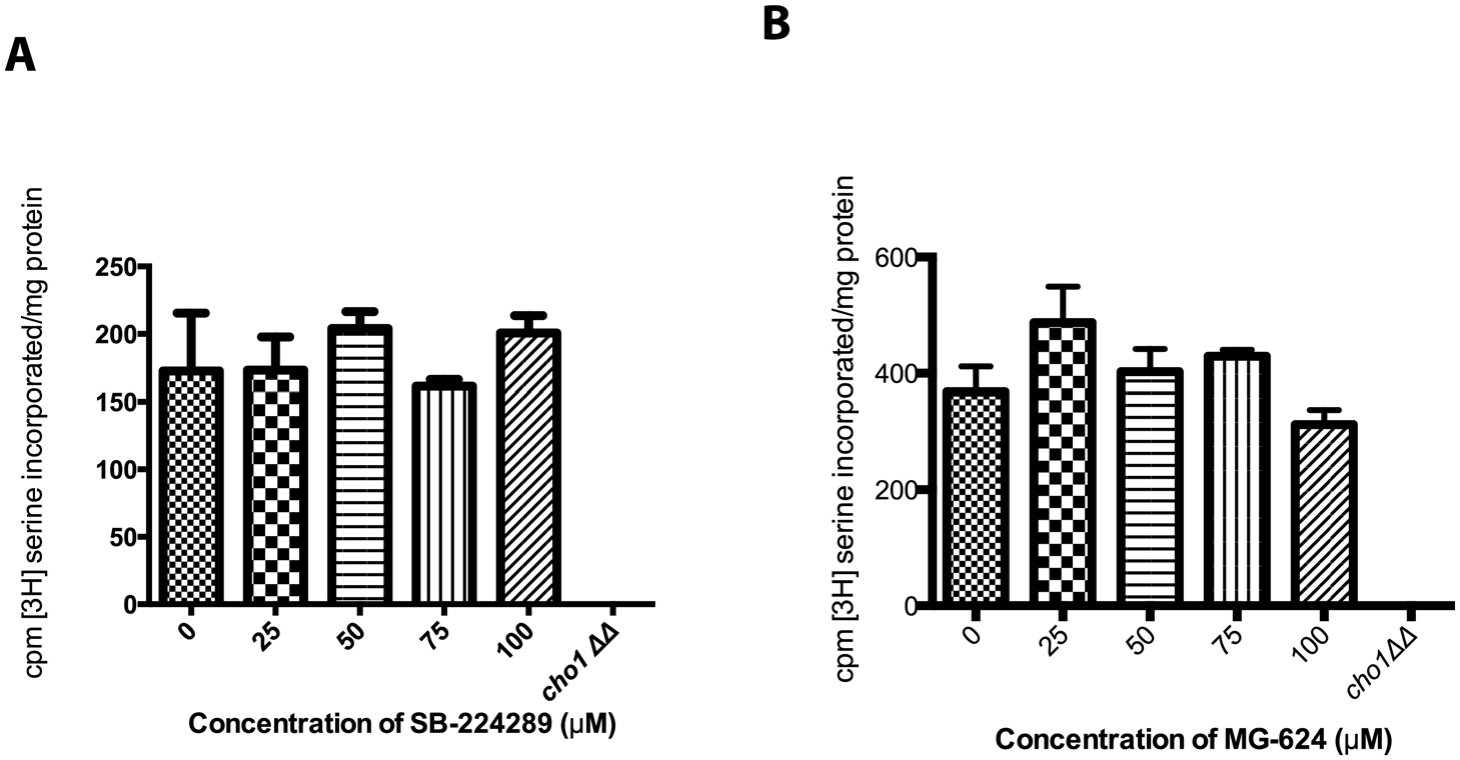
SB-224289 and MG-624 Do Not Inhibit the Activity of Cho1p. PS synthase activity shown as counts per minute per milligram of protein, quantifying ^3^H-l-serine incorporated into PS. Addition of varying concentrations of SB-224289 (A) and MG-624 (B) did not have an inhibitory effect on PS production.

We hypothesized that SB-224289 inhibited the PS synthase enzyme, Cho1p, thus reducing levels of PS and allowing wild-type cells to survive despite the activity of Pap-A. In order to test this hypothesis, we performed a PS synthase assay to assess the activity of Cho1p with the addition of varying concentrations of SB-224289. Upon addition of SB-224289, no changes were seen in the activity of the Cho1p enzyme as compared to the wild-type control. This indicated that SB-224289 does not directly inhibit the PS synthase enzyme in this *in vitro* assay (Fig 5A). Furthermore, a PS synthase assay was performed with MG-624 at varying concentrations. Again, no PS synthase inhibition was observed in any concentration tested (Fig 5B).

To further investigate the mode of action of SB-224289, we assessed the compound’s ability to inhibit PS synthesis *in vivo*. If the compound must be metabolized or modified internally in order to inhibit Cho1p, we should see a decrease in PS levels only when treating live cells with SB-224289. Previous studies showed that *cho1ΔΔ*, which has no PS, shows a distinct phenotype of β (1–3)-glucan unmasking [25]. Based on this, we performed an assay to determine if treatment with SB-224289 would cause the β (1–3)-glucan in the cell wall to become exposed. Cells were treated with SB-224289 for 3 hours and then stained with β (1–3)-glucan antibody. However, epifluorescence microscopy showed no indication that β (1–3)-glucan was exposed in SB-224289-treated cells. PS is also a precursor for the synthesis of phosphatidylethanolamine (PE), in a pathway where PS is decarboxylated to form PE. As a result, the *cho1ΔΔ* mutant, since it lacks PS, requires ethanolamine in the medium so it can make PE from imported ethanolamine via the Kennedy pathway. If SB-224289 is a PS synthase inhibitor, then it should have caused ethanolamine auxotrophy in wild-type *C*. *albicans*, however, this was not the case.

### SB-224289 specifically blocks the activity of papuamides and not other membrane disruptors

**Fig 6:**
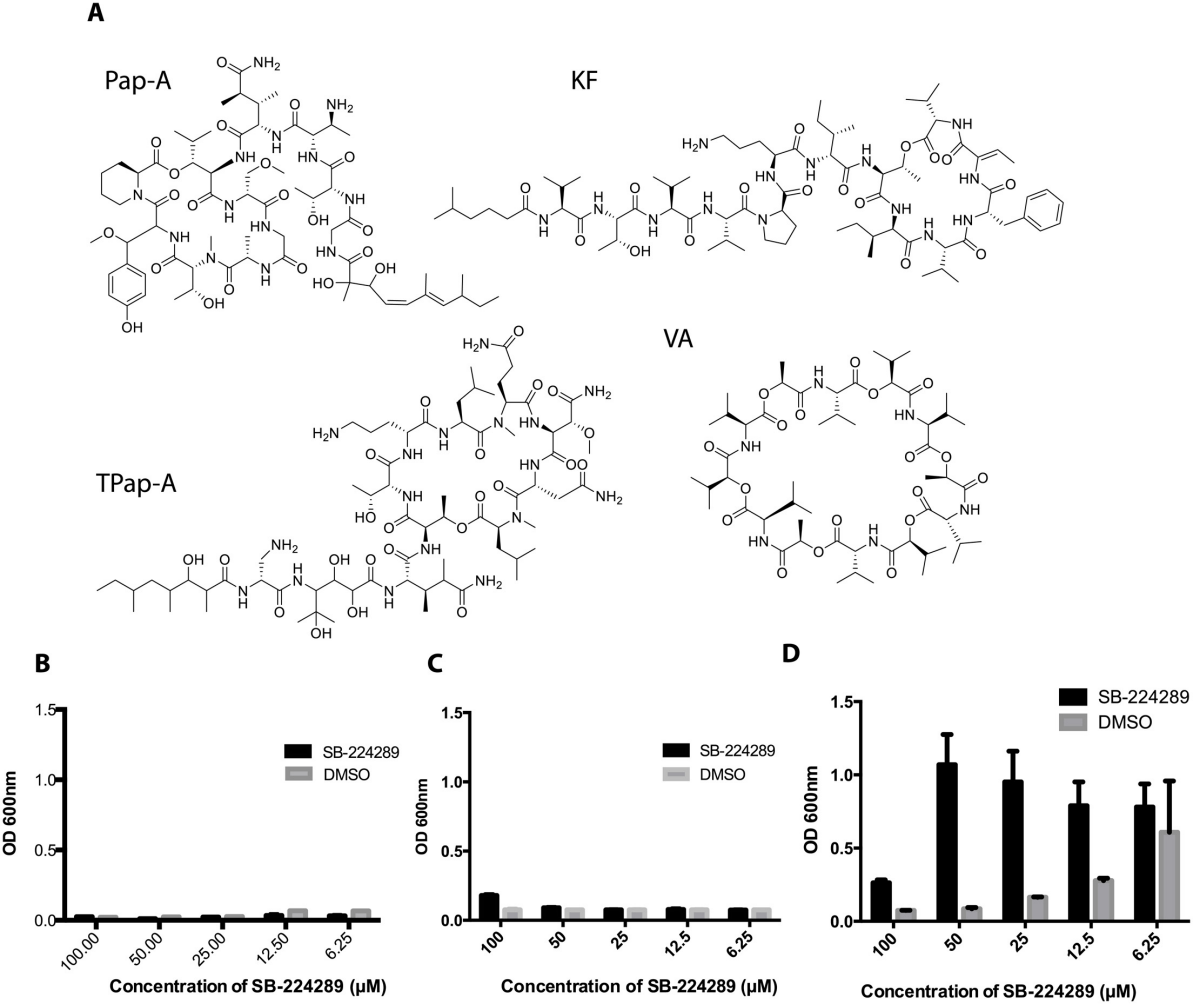
SB-224289 Protects Against Theopapuamide, but Not Other Depsipeptides. **A.** The structure of several depsipeptides are shown. For each of these compounds, 1x10^4^ cells/ml were treated with serially diluted concentrations of SB-224289 (SB) and were incubated for 3 hours at 37°C. Following this incubation, **B.** 3 μg/ml valinomycin (VA), **C**. 15 μg/ml kahalalide F (KF), or **D**. 6 μg/ml theopapuamide (TA) was added and further allowed to incubate overnight. Optical density (OD) was read at 600 nm the next day as a measure of cell survival.

Naturally-produced depsipeptides share similar structures (Fig 6A) and can be medically relevant like the antibiotic valinomycin (VA) produced from *Streptomyces spp*. [26, 27], the emetic toxin cereulide produced by the foodborne pathogen *Bacillus cereus* [27–30], and anti-tumor drug prospect kahalalide F (KF) found in sea slugs [31]. A previous study found that a serotonin receptor antagonist, ondansetron hydrochloride, abolished the emetic effect of cereulide in Asian house shrews [30]. Theopapuamide A (TPap-A) is a close structural relative of Pap-A and was previously shown to have potent antifungal activity [32, 33]. Based on these studies, we were interested in determining if SB-224289 would block the activities of similar depsipeptide toxins.

First, we tested if SB-224289 could rescue cells from toxic levels of VA. We treated *C*. *albicans* with serially diluted concentrations of SB-224289, followed by treatment with 3 μg/ml of VA. We found that no concentration of SB-224289 was sufficient to provide protection to cells (Fig 6B). We further measured the ability of SB-224289 to mitigate the toxicity of KF and TPap-A (Fig 6C and 6D). SB-224289 was unable to protect wild-type cells against KF, but we found that SB-224289 was able to provide protection against TPap-A.

## Discussion

The screen described in this communication was originally intended to identify inhibitors of the fungal PS synthase. However, the identified hit compound, SB-224289, does not inhibit PS synthesis even though it interferes with the lethal effects of Pap-A on *C*. *albicans* cells that contain PS. The evidence that SB-224289 does not block PS synthesis comes from several secondary screens based on the *cho1ΔΔ* mutant’s characteristic phenotypes [13]. For example, the *cho1ΔΔ* mutant is an ethanolamine auxotroph because *C*. *albicans* uses PS as a precursor for production of PE, a vital phospholipid, in one of its two PE synthesis pathways. Thus, ethanolamine must be supplemented in the medium so that the *cho1*ΔΔ mutant can produce PE through the alternative Kennedy Pathway. We saw no indication that treatment with SB-224289 caused wild-type *C*. *albicans* to become an ethanolamine auxotroph, indicating that it does not interfere with PS synthesis. A second useful phenotype is increased exposure of β (1–3)-glucan in the cell wall upon loss of PS. The cause of this exposure in *cho1ΔΔ* is currently unknown, but it is very specific to the loss of PS rather than PE [25]. As with the previous assay, treatment with SB-224289 does not lead to exposure of β (1–3)-glucan, thus indicating that it does not interfere with PS synthesis. Finally, SB-224289 did not interfere with PS synthesis in an *in vitro* assay (Fig 5A).

The most interesting aspect of the blockade of the lethal effects of Pap-A on *C*. *albicans* by SB-224289 is its specificity. Seven different analogs representing fragments of SB-224289 or the entire molecule fail to rescue *C*. *albicans* from Pap-A-induced cytotoxicity. These results indicate that the activity of SB-224289 is specific and relies on the entire structure of the molecule (Fig 4), possibly, due to a specific three-dimensional conformation which could only be adopted by the full SB-224289 molecule. In addition, SB-224289 could only inhibit the toxicity of depsipeptides that were structurally highly similar. For example, we saw no indication that SB-224289 provided protection to wild-type *C*. *albicans* treated with VA or KF (Fig 6). However, as expected, SB-224289 was able to abrogate the cytotoxicity of TPap-A, a close structural relative of Pap-A (Fig 6) likely to bind PS as well. Altogether, these data suggest that there might be a physical interaction between the two compounds that inhibits the toxic effects of Pap-A. More specifically, structural differences in the tail regions of these related depsipeptides may confer their selectivity. It is also possible that SB-224289 impacts cellular physiology in a way that creates a protective response, but it is unclear what such a mechanism might entail.

Further studies are needed to understand the nature of the interaction between SB-224289 and Pap-A and the mechanism that leads to protection of *C*. *albicans* cells against Pap-A toxicity. Although SB-224289 is clearly not acting by inhibiting Cho1p, the selective inactivation of the Pap-A toxin by SB-224289 indicates a path to the discovery of other compounds that can inactivate similar membrane active toxins. In fact, the assay described herein could be easily adapted from use with Pap-A to any number of membrane potent toxins to find antagonists. The compounds discovered by this approach would be useful for treatments against environmental toxins that occur during algal blooms that affect drinking water, food contamination, etc. It could also be useful as a method for identifying potential drug interactions that would counter-indicate compounds or combination therapies being considered as potential anti-cancer or anti-microbial drugs.

## Supporting Information

S1 File Synthesis of SB-224289 fragments. Detailed scheme for synthesis of fragments of SB-224289.
(DOCX)
