# Supplementary material for: SB-224289 Antagonizes the Antifungal Mechanism of the Marine Depsipeptide Papuamide A
Source: PLoS One. 2016 May 16;11(5):e0154932. doi: 10.1371/journal.pone.0154932 (PMC4868317; doi:10.1371/journal.pone.0154932)
Supplement: S1 File — Detailed scheme for synthesis of fragments of SB-224289. (DOCX) [file pone.0154932.s001.docx]

**S1 File**

**SB-224289 antagonizes the antifungal mechanism of the marine depsipeptide Papuamide A**

Chelsi D. Cassilly,^1^ Marcus M. Maddox,^2^ Philip T. Cherian,^2^ John J. Bowling,^2^ Mark T. Hamann,^3^ Richard E. Lee,^2^ Todd B. Reynolds^1^*

1. Department of Microbiology, University of Tennessee, Knoxville, TN, USA

2. Department of Chemical Biology and Therapeutics, St. Jude Children’s Research Hospital, Memphis, TN, USA

3. Department of Drug Discovery and Biomedical Sciences, College of Pharmacy, Medical University of South Carolina, Medical University of South Carolina, Charleston, SC, USA

Corresponding Author: TR, [treynol6@utk.edu](mailto:treynol6@utk.edu)

Coauthors Email (for submission): CC, [ccassill@utk.edu](mailto:ccassill@utk.edu); MM,mmaddox6@uthsc.edu; PC, philip.cherian@stjude.org; JB, [john.bowling@stjude.org](mailto:john.bowling@stjude.org); MH, [hamannm@musc.edu](mailto:hamannm@musc.edu); RL, richard.lee@stjude.org

**Table of contents**

| **Title** | **Page** |
| --- | --- |
| Synthesis of compounds | 3 |
| References | 10 |

**Synthesis of SB224289 fragments**

**General methods**: All reagents and solvents were purchased from commercial sources and were used without further purification. The final reaction mixtures were purified on a Biotage Isolera Flash Purification System. The purity and mass of the synthesized compounds were determined on a Waters ACQUITY UPLC-PDA-ELSD-MS system using C_18_ reverse phase column and 0.1% formic acid/water - 0.1% formic acid/acetonitrile as solvents. All synthesized compounds were at least 95% pure. The structures of the synthesized compounds were confirmed by ^1^H and ^13^C NMR that were recorded on either 400 MHz Varian AVANCE 400- FT NMR or 500 MHz Bruker AVANCE III HD NMR.

**Synthesis of 1-acetylindolin-5-yl acetate** [[1](#_ENREF_1), [2](#_ENREF_2)]

To 1,1'-(indoline-1,5-diyl)diethanone (203 mg, 1 mmol) were added glacial acetic acid (2002 µl, 35.0 mmol). The mixture was stirred for a few minutes to afford a clear solution. Next, 400μl of peracetic acid (121 µl, 1.8 mmol) (32% solution in dilute acetic acid) were added to the reaction and the mixture was allowed to stand at room temperature for 20h. UPLC indicates formation of product. 20ml water was added to the reaction mixture and the aqueous layer was extracted with EtOAc (20ml x 3). The combined EtOAc layers were washed sequentially with water, saturated NaHSO_3_, 5% Na_2_CO_3_, brine, dried over Na_2_SO_4_ and concentrated to provide 190 mg (87%) of off-white solid. ^1^H NMR (500 MHz, CDCl3) δ 2.22 (s, 3H), 2.28 (s, 3H), 3.20 (t, *J* = 8.5 Hz, 2H), 4.08 (t, *J* = 8.5 Hz, 2H), 6.88 (dd, *J* = 8.7, 2.3 Hz, 1H), 6.92 (s, 1H), 8.20 (d, *J* = 8.7 Hz, 1H). ^13^C NMR (126 MHz, CDCl3) δ 21.09, 24.08, 27.93, 49.05, 117.37, 118.05, 120.29, 132.41, 140.73, 146.52, 168.52, 169.84. ESI-MS: calc. for C_12_H_13_NO_3_ [M+H]^+^: 219.24; found: 219.83

**Synthesis of 1-(6-bromo-5-hydroxyindolin-1-yl)ethan-1-one** [[1](#_ENREF_1)]

The 1-acetylindolin-5-yl acetate (1.096g, 5mmol) was dissolved in 25ml of MeOH and 10ml of a 1M solution of sodium hydroxide (0.400 g, 10 mmol) were added and the mixture stirred for 4h. The MeOH were evaporated and the aqueous layer acidified with aqueous 5% HCl. The precipitate was filtered, washed with water and dried under vacuum to provide 0.713g (80%) of 1-(5-hydroxyindolin-1-yl)ethanone as light brown solid which was used in next reaction as is. The solid was dissolved in 25ml acetic acid and 1-bromopyrrolidine-2,5-dione (716 mg, 4.02 mmol) were added. After stirring for 1h, the reaction mixture was cooled in an ice bath and 200ml of ice cold water were added. The precipitated product was filtered, washed with water and dried under Vacuum overnight to provide 685mg (67%) of 1-(6-bromo-5-hydroxyindolin-1-yl)ethan-1-one as brown solid. ^1^H NMR (500 MHz, DMSO) δ 2.11 (s, 3H), 3.05 (t, J = 8.4 Hz, 2H), 4.05 (t, J = 8.5 Hz, 2H), 6.83 (s, 1H), 8.14 (s, 1H), 9.89 (s, 1H). ^13^C NMR (126 MHz, DMSO) δ 24.08, 27.72, 48.85, 106.61, 113.18, 119.82, 133.34, 136.55, 150.29, 168.28. ESI-MS: calc. for C_10_H_10_BrNO_2_ [M+H]^+^: 256.09; found: 255.99.

**Synthesis of (1-methyl-1,2,3,6-tetrahydropyridin-4-yl)methanol** [[3](#_ENREF_3)]

A mixture of pyridin-4-ylmethanol (2.000 g, 18.33 mmol) and iodomethane (1.284 ml, 20.62 mmol) under N_2_ was heated at 120°c for 3h. After cooling to room temperature, 30ml of dry MeOH were added and the solution cooled in an ice bath. Then NaBH_4_ (2.080 g, 55mmol) were added in three portions over a period of 20mins and the mixture stirred at room temperature for 3h. The solvent was evaporated and the residue treated with 20ml of ice cold water. The aqueous layer was extracted with chloroform (20ml x 5). The aqueous layer was then basified with 5% NaOH and extracted again with chloroform (20ml x 5). The combined chloroform layers were dried over Na_2_SO_4_ and concentrated to provide 1.62g (70%) of (1-methyl-1,2,3,6-tetrahydropyridin-4-yl)methanol as orange oil. ^1^H NMR (500 MHz, CD_3_CN) δ 2.02 – 2.10 (m, 2H), 2.25 (s, 3H), 2.45 (t, J = 5.8 Hz, 2H), 2.83 (h, J = 2.2 Hz, 2H), 3.87 (s, 2H), 5.54 – 5.56 (m, 1H). ^13^C NMR (126 MHz, CD_3_CN) δ 26.86, 45.61, 52.31, 54.60, 65.56, 119.68, 136.94. ESI-MS: calc. for C_7_H_13_NO [M+H]^+^: 127.18; found: 127.65.

**Synthesis of 1-(6-bromo-5-((1-methyl-1,2,3,6-tetrahydropyridin-4-yl)methoxy)indolin-1-yl)ethanone** [[4](#_ENREF_4)]

To 10ml dry THF were added (1-methyl-1,2,3,6-tetrahydropyridin-4-yl)methanol (653 µl, 4.94 mmol) followed by 1-(6-bromo-5-hydroxyindolin-1-yl)ethanone (1264 mg, 4.94 mmol) and triphenylphosphine (1683 mg, 6.42 mmol). The suspension was cooled in an ice bath and (E)-diethyl diazene-1,2-dicarboxylate (1162 µl, 7.40 mmol) were added dropwise. The ice bath was removed and the mixture stirred overnight. UPLC indicates 50% of the indoline is unreacted. Hence an additional 0.5 eq of all other reagents was added and the mixture stirred for 24h. The solvent was evaporated and the residue dissolved in 25ml EtOAc. The EtOAc layer was then extracted with 5% HCl (20ml x 2). The combined HCl layers were treated dropwise with 2N NaOH till the pH was ~8. The obtained solid was then filtered, washed with water and dried under vacuum to provide 1g (55%) of 1-(6-bromo-5-((1-methyl-1,2,3,6-tetrahydropyridin-4-yl)methoxy)indolin-1-yl)ethanone as brown solid. ^1^H NMR (500 MHz, CDCl_3_) δ 2.20 (s, 3H), 2.31 (s, 2H), 2.37 (s, 3H), 2.60 (t, J = 5.5 Hz, 2H), 2.99 (s, 2H), 3.14 (t, J = 8.3 Hz, 2H), 4.06 (t, J = 8.3 Hz, 2H), 4.44 (s, 2H), 5.80 (s, 1H), 6.75 (s, 1H), 8.42 (s, 1H). ^13^C NMR (126 MHz, CDCl_3_) δ 23.83, 26.34, 28.11, 45.53, 49.09, 51.68, 54.05, 72.66, 110.48, 110.73, 121.48, 122.40, 131.52, 131.79, 137.33, 151.50, 168.37. ESI-MS: calc. for C_7_H_21_BrN_2_O_2_ [M+H]^+^: 365.26; found: 365.13.

**Synthesis of 1-(1'-methyl-6,7-dihydrospiro[furo[2,3-f]indole-3,4'-piperidin]-5(2H)-yl)ethanone (2945)** [[4](#_ENREF_4)]

A mixture of 1-(6-bromo-5-((1-methyl-1,2,3,6-tetrahydropyridin-4-yl)methoxy)indolin-1-yl)ethanone (208 mg, 0.569 mmol) and catalytic amount of AIBN (E)-2,2'-(diazene-1,2-diyl)bis(2-methylpropanenitrile) (8.21 mg, 0.05 mmol) under N_2_ was heated to reflux in 8ml of anhydrous benzene. Next, tributylstannane (230 µl, 0.854 mmol) in 3ml of anhydrous benzene was added dropwise and the mixture refluxed for additional 6h. The benzene was evaporated and the residue treated with 10ml 5% HCl and 8ml EtOAc. The mixture was shaken vigorously and the two layers separated. The HCl layer was then basified with 2N NaOH and the precipitated was collected and purified by column chromatography (5% MeOH/DCM to 10% MeOH/DCM) to provide 85mg (52%) of **2945** as off-white solid. ^1^H NMR (400 MHz, CDCl3) δ 1.69 – 1.80 (m, 2H), 1.96 – 2.11 (m, 4H), 2.19 (s, 3H), 2.33 (s, 3H), 2.86 (d, J = 8.8 Hz, 2H), 3.12 (t, J = 8.4 Hz, 2H), 4.03 (t, J = 8.4 Hz, 2H), 4.36 (s, 2H), 6.61 (s, 1H), 8.12 (s, 1H). ^13^C NMR (101 MHz, CDCl3) δ 23.97, 28.13, 36.31, 44.00, 46.38, 49.25, 53.01, 81.13, 106.06, 112.30, 131.68, 133.71, 136.88, 155.83, 167.83. ESI-MS: calc. for C_7_H_22_N_2_O_2_ [M+H]^+^: 286.37; found: 286.80.

**Synthesis of 1'-methyl-2,5,6,7-tetrahydrospiro[furo[2,3-f]indole-3,4'-piperidine] (2946)** [[4](#_ENREF_4)]

To 1-(1'-methyl-6,7-dihydrospiro[furo[2,3-f]indole-3,4'-piperidin]-5(2H)-yl)ethanone (130 mg, 0.454 mmol) were added 10 ml of 5M HCl and 5ml of EtOH and the mixture refluxed for 2h and then stirred at room temperature for 16h. The EtOH was evaporated and the remaining solution was cooled in an ice bath. The solution was treated with 40% NaOH till the pH was ~12. The obtained precipitate was filtered, washed with water and dried under vacuum to provide 76mg (69%) of **2946** as beige solid. ^1^H NMR (400 MHz, CDCl_3_) δ 1.68 – 1.76 (m, 2H), 1.96 (t, J = 9.1 Hz, 4H), 2.31 (s, 3H), 2.83 (d, J = 7.1 Hz, 2H), 2.96 (t, J = 8.1 Hz, 2H), 3.52 (t, J = 7.9 Hz, 3H), 4.31 (s, 2H), 6.47 (s, 1H), 6.61 (s, 1H). ^13^C NMR (101 MHz, CDCl_3_) δ 30.47, 36.41, 43.99, 46.50, 48.03, 53.17, 80.40, 104.92, 106.60, 129.95, 133.39, 145.36, 152.99. ESI-MS: calc. for C_15_H_20_N_2_O [M+H]^+^: 244.33; found: 244.94.

**Synthesis of (1'-methyl-6,7-dihydrospiro[furo[2,3-f]indole-3,4'-piperidin]-5(2H)-yl)(phenyl)methanone (3047)**

To a cold solution of 1'-methyl-2,5,6,7-tetrahydrospiro[furo[2,3-f]indole-3,4'-piperidine] (25.00 mg, 0.102 mmol) and triethylamine (17.11 µl, 0.123 mmol) in 3ml of dry DCM under N_2_, were added benzoyl chloride (13.08 µl, 0.113 mmol). The ice bath was removed and the mixture stirred at room temperature for 4h. UPLC indicates formation of product. The DCM layer was extracted with water, brine, dried over Na_2_SO_4_ and concentrated. The crude was purified by reverse phase chromatography to provide 25mg (70%) of **3047** as colorless oil. ^1^H NMR (500 MHz, CDCl_3_) δ 1.60 – 1.80 (br s, 2H), 1.90 – 2.15 (br s, 4H), 2.32 (s, 3H), 2.66 – 2.94 (br s, 2H), 3.03 (t, J = 7.9 Hz, 2H), 3.90 – 4.18 (m, 2H), 4.37 (s, 2H), 6.64 (s, 1H), 7.38 – 7.48 (m, 3H), 7.53 (d, J = 7.5 Hz, 2H), 8.12 (s, 1H). ^13^C NMR (126 MHz, CDCl_3_) δ 28.66, 36.39, 44.00, 46.41, 51.66, 52.96, 67.96, 81.11, 106.25, 113.15, 126.90, 128.50, 129.99, 132.81, 133.85, 136.58, 137.42, 156.32, 168.25. ESI-MS: calc. for C_22_H_24_N_2_O_2_ [M+H]^+^: 348.43; found: 348.65.

**Synthesis of (1'-methyl-6,7-dihydrospiro[furo[2,3-f]indole-3,4'-piperidin]-5(2H)-yl)(2'-methyl-[1,1'-biphenyl]-4-yl)methanone (3048)**

To a cold solution of 2'-methyl-[1,1'-biphenyl]-4-carboxylic acid (23.89 mg, 0.113 mmol) in 2ml of dry DCM containing two drops of DMF were added oxalyl dichloride (11.41 µl, 0.133 mmol) and the mixture was stirred at room temperature for 2h. The DCM was evaporated and the residue redissolved into 1ml DCM and transferred dropwisely to another round bottom flask containing 1'-methyl-2,5,6,7-tetrahydrospiro[furo[2,3-f]indole-3,4'-piperidine] (25.00 mg, 0.102 mmol) and triethylamine (28.5 µl, 0.205 mmol) in 3ml of cold dry DCM. After stirring for 4h at room temperature the DCM layer was extracted with water, brine, dried over Na_2_SO_4_ and concentrated. The crude was purified by RPCC to provide 22mg (49%) of **3047** as yellow oil. 1H NMR (400 MHz, CDCl_3_) δ 1.65 – 1.85 (br s, 2H), 1.90 – 2.15 (br s, 4H), 2.29 (s, 4H), 2.32 (s, 3H), 2.88 (s, 2H), 3.06 (t, J = 8.1 Hz, 2H), 4.02 – 4.22 (br s, 2H), 4.31 – 4.44 (br s, 2H), 6.66 (s, 1H), 7.21 – 7.30 (m, 4H), 7.39 (d, J = 8.1 Hz, 2H), 7.59 (d, J = 8.0 Hz, 2H), 8.15 (s, 1H). ^13^C NMR (101 MHz, CDCl_3_) δ 20.44, 28.73, 36.32, 43.93, 46.36, 51.72, 52.92, 81.14, 106.26, 113.25, 125.86, 126.94, 127.64, 129.33, 129.67, 130.43, 135.26, 141.02, 156.34, 168.18. ESI-MS: calc. for C_22_H_24_N_2_O_2_ [M+H]^+^: 438.56; found: 438.97.

**References**

1. Wyman PA, Marshall HR, Flynn ST, King RJ, Thompson M, Smith PW, et al. Identification of a potent and selective 5-HT1B receptor antagonist. Bioorganic & Medicinal Chemistry Letters. 2005;15(21):4708-12. doi: <http://dx.doi.org/10.1016/j.bmcl.2005.07.085>.

2. Patel A, Liebner F, Netscher T, Mereiter K, Rosenau T. Vitamin E Chemistry. Nitration of Non-α-tocopherols:  Products and Mechanistic Considerations. The Journal of Organic Chemistry. 2007;72(17):6504-12. doi: 10.1021/jo0706832.

3. Gijsen HJM, De Cleyn MJA, Love CJ, Surkyn M, Van Brandt SFA, Verdonck MGC, et al. Development of two diastereoselective routes towards trans-4-aminomethyl-piperidin-3-ol building blocks. Tetrahedron. 2008;64(10):2456-64. doi: <http://dx.doi.org/10.1016/j.tet.2007.12.055>.

4. Gaster LM, Blaney FE, Davies S, Duckworth DM, Ham P, Jenkins S, et al. The Selective 5-HT1B Receptor Inverse Agonist 1‘-Methyl-5-[[2‘-methyl-4‘- (5-methyl-1,2,4-oxadiazol-3-yl)biphenyl-4-yl]carbonyl]-2,3,6,7-tetrahydro- spiro[furo[2,3-f]indole-3,4‘-piperidine] (SB-224289) Potently Blocks Terminal 5-HT Autoreceptor Function Both in Vitro and in Vivo. Journal of Medicinal Chemistry. 1998;41(8):1218-35. doi: 10.1021/jm970457s.
